# Supplementary material for: The yeast endocytic early/sorting compartment exists as an independent sub-compartment within the trans-Golgi network
Source: eLife. 2023 Jul 21;12:e84850. doi: 10.7554/eLife.84850 (PMC10361720; doi:10.7554/eLife.84850)
Supplement: Supplementary file 1. [file elife-84850-supp1.doc]

**Table S1. Yeast strains**

Strain Genotype Source

JJTY00501 *Mat***a** *his3*Δ*1* *leu2*Δ*0 ura3*Δ*0* *lys2*Δ*0 bar1*Δ::*LEU2* Toshima lab

JJTY01960 *Mat***a** *his3*-Δ*200* *leu2-3*, *112* *ura3-52 lys2-801 GFP-VPS21*::*HIS3 ABP1-mCherry*::*URA3* This study

JJTY04630 *Mat***a** *his3*Δ*1* *leu2*Δ*0* *ura3*Δ*0* *lys2*Δ*0 ypt32*Δ::*LEU2 GFP-YPT31*::*HIS3* This study

JJTY04680 *Mat***a** *his3*Δ*1* *leu2*Δ*0* *ura3*Δ*0* *lys2*Δ*0 bar1*Δ::*LEU2 ypt32*Δ::*LEU2* This study

JJTY05187 *Mat***a** *his3*Δ*1* *leu2*Δ*0* *ura3*Δ*0* *lys2*Δ*0 bar1*Δ::*LEU2 GFP-YPT31*::*HIS3* This study

JJTY05510 *Mat***a** *his3*Δ*1* *leu2*Δ*0* *ura3*Δ*0* *lys2*Δ*0 GFP-VPS21*::*HIS3 SEC7-mCherry*::*URA3* This study

JJTY06538 *Mat***a** *his3*Δ*1 leu2*Δ*0 ura3*Δ*0 met15*Δ*0 ent3*Δ::*KanMX6 ent5*Δ::*LEU2 bar1*Δ::*URA3* Toshima lab

JJTY07143 *Mat***a** *his3*Δ*1* *leu2*Δ*0 ura3*Δ*0* *lys2*Δ*0 apl4*Δ::*KanMX bar1*Δ::*LEU2* Toshima lab

JJTY07979 *Mat***α** *his3*-Δ*200, leu2-3*, *112* *ura3-52 lys2-801, ABP1-mCherry*::*URA3 sla1Δ::LEU2* Toshima lab

JJTY08610 *Mat***a** *his3*Δ*1* *leu2*Δ*0* *ura3*Δ*0* *lys2*Δ*0 gga1*Δ::*KanMX6 gga2*Δ::*URA3 bar1*Δ::*LEU2* This study

JJTY08751 *Mat***a** *his3*Δ*1* *leu2*Δ*0 ura3*Δ*0* *lys2*Δ*0 gga1*Δ::*KanMX6 gga2*Δ::*URA bar1*Δ::*LEU2*

*GFP-TLG2*::*HIS3* This study

JJTY08752 *Mat***a** *his3*Δ*1* *leu2*Δ*0 ura3*Δ*0* *lys2*Δ*0 gga1*Δ::*KanMX6 gga2*Δ::*URA bar1*Δ::*LEU2*

*SEC7-GFP*::*HIS3* This study

JJTY08753 *Mat***a** *his3*Δ*1 leu2*Δ*0* *ura3*Δ*0* *lys2*Δ*0 met15*Δ*0 ent3*Δ::*KanMX6 ent5*Δ::*URA bar1*Δ::*LEU2*

*SEC7-GFP*::*HIS3* This study

JJTY08755 *Mat***a** *his3*-Δ*200* *leu2-3*, *112* *ura3-52* *bar1*Δ::*LEU2 SEC7-GFP*::*HIS3* This study

JJTY08756 *Mat***a** *his3*-Δ*200* *leu2-3*, *112* *ura3-52 bar1*Δ::*LEU2 GFP-TLG1*::*HIS3* This study

JJTY08757 *Mat***a** *his3*-Δ*200* *leu2-3*, *112* *ura3-52 bar1*Δ::*LEU2 GFP-TLG2*::*HIS3* This study

JJTY08759 *Mat***a** *his3*Δ*1* *leu2*Δ*0 ura3*Δ*0* *lys2*Δ*0 bar1*Δ::*LEU2 SEC7-mCherry*::*URA GFP-TLG2*::*HIS3* This study

JJTY08760 *Mat***a** *his3*-Δ*200* *leu2-3*, *112* *ura3-52* *bar1*Δ::*LEU2 GFP-SNC1*::*HIS3* This study

JJTY08969 *Mat***a** *his3*Δ*1* *leu2*Δ*0* *ura3*Δ*0* *lys2*Δ*0 gga1*Δ::*KanMX6 gga2*Δ::*URA3 bar1*Δ::*LEU2 GFP-TLG2*::*HIS3 SEC7-mCherry*::*HphMX4* This study

JJTY08972 *Mat***a** *his3*-Δ*200* *leu2-3*, *112* *ura3-52 bar1*Δ::*LEU2 GFP-TLG2*::*HIS3 GGA2-mCherry*::*URA3* This study

JJTY08974 *Mat***a** *his3*-Δ*200* *leu2-3*, *112* *ura3-52 bar1*Δ::*LEU2 GFP-TLG2*::*HIS3 mCherry-VPS21*::*URA3* This study

JJTY10261 *Mat***a** *his3*Δ*1* *leu2*Δ*0* *ura3*Δ*0* *met15*Δ*0 gga1*Δ::*KanMX6 gga2*Δ::*KanMX6 GFP-TLG2*::*HIS3* This study

*mCherry-SNC1*::*LEU*

JJTY10262 *Mat***a** *his3-*Δ*200 leu2-3, 112 ura3-52 lys2-801 vps21*Δ::*KanMX6 ypt52*Δ::*KanMX6 bar1*Δ::*LEU2*

*GFP-TLG2*::*HIS3* This study

JJTY10263 *Mat***a** *his3-*Δ*200 leu2-3, 112 ura3-52 lys2-801 mCherry-SNC1*::*LEU2 GFP-TLG2*::*HIS3* This study

JJTY10267 *Mat***a** *his3*-Δ*200* *leu2-3*, *112* *ura3-52 bar1*Δ::*LEU2 GFP-TLG2*::*HIS3 ABP1-mCherry*::*URA3* This study

JJTY10271 *Mat***a** *his3*-Δ*200* *leu2-3*, *112* *ura3-52 arp3D11A*::*LEU2 ABP1-mCherry*::*URA3 GFP-TLG2*::*HIS3* This study

JJTY11230 *Mat***a** *his3*-Δ*200* *leu2-3*, *112* *ura3-52 bar1*Δ::*LEU2 GFP-TLG2*::*HIS3*

[pRS316-*ADH1p-SEC7-iRFP*::*URA3*] 　　　　 This study

JJTY11231 *Mat***a** *his3*Δ*1* *leu2*Δ*0 ura3*Δ*0* *lys2*Δ*0 gga1*Δ::*KanMX6 gga2*Δ::*KanMX6 bar1*Δ::*LEU2*

*GFP-TLG2*::*HIS3*  [pRS316-*ADH1p-SEC7-iRFP*::*URA3*] This study

JJTY11233 *Mat***a** *his3*-Δ*200* *leu2-3*, *112* *ura3-52 GFP-TLG2*::*HIS3 ABP1-mCherry*::*LEU2*

[pRS316-*ADH1p-SEC7-iRFP*::*URA3*] This study

JJTY11235 *Mat***a** *his3*-Δ*200* *leu2-3*, *112* *ura3-52 GFP-TLG2*::*HIS3 mCherry-VPS21*::*URA3*

[pRS315-*ADH1p-SEC7-iRFP*::*LEU2*] This study

JJTY11239 *Mat***a** *his3*Δ*1* *leu2*Δ*0 ura3*Δ*0* *lys2*Δ*0 GFP-TLG2*::*HIS3 GGA2-mCherry*::*LEU2*

[pRS316-*ADH1p-SEC7-iRFP*::*URA3*] This study

JJTY11240 *Mat***a** *his3*Δ*1* *leu2*Δ*0 ura3*Δ*0* *lys2*Δ*0 GFP-TLG2*::*HIS3 mCherry-SNC1*::*LEU2*

[pRS316-*ADH1p-SEC7-iRFP*::*URA3*] This study

JJTY11241 *Mat***a** *his3*Δ*1* *leu2*Δ*0 ura3*Δ*0* *lys2*Δ*0 gga1*Δ::*KanMX6 gga2*Δ::*KanMX6 GFP-TLG2*::*HIS3*

*mCherry-SNC1*::*LEU2* [pRS316-*ADH1p-SEC7-iRFP*::*URA3*] This study

JJTY11242 *Mat***a** *his3*Δ*1* *leu2*Δ*0 ura3*Δ*0* *rcy1*Δ::*KanMX6 GFP-TLG2*::*HIS3 mCherry-SNC1*::*LEU2*

[pRS316-*ADH1p-SEC7-iRFP*::*URA3*] This study

JJTY11255 *Mat***a** *his3*-Δ*200* *leu2-3*, *112* *ura3-52 bar1*Δ::*LEU2GFP-TLG1*::*HIS3 mCherry-TLG2*::*URA3* This study

JJTY11257 *Mat***a** *his3*Δ*0 leu2*Δ*0* *ura3*Δ*0* *lys2*Δ*0 met15*Δ*0 gga1*Δ::*KanMX6 gga2*Δ::*KanMX6* *bar1*Δ::*LEU2*

*GFP-TLG1*::*HIS3 mCherry-TLG2*::*URA3* This study

JJTY11269 *Mat***a** *his3*-Δ*200* *leu2-3*, *112* *ura3-52 GFP-TLG2*::*HIS3 mCherry-YPT31*::*LEU2* This study

[pRS316-*ADH1p-SEC7-iRFP*::*URA3*]

JJTY11273 *Mat***a** *his3*-Δ*200* *leu2-3*, *112* *ura3-52 GFP-YPT31*::*HIS3 mCherry-SNC1*::*LEU2* This study

[pRS316-*ADH1p-SEC7-iRFP*::*URA3*]

JJTY11276 *Mat***a** *his3*Δ*1* *leu2*Δ*0 ura3*Δ*0* *lys2*Δ*0 met15*Δ0 *gga1*Δ::*KanMX6 gga2*Δ::*KanMX6* This study

*GFP-YPT31*::*HIS3 mCherry-SNC1*::*LEU2* [pRS316-*ADH1p-SEC7-iRFP*::*URA3*]

JJTY11277 *Mat***a** *his3*Δ*1* *leu2*Δ*0 ura3*Δ*0* *lys2*Δ*0 GFP-YPT31*::*HIS3 mCherry-YPT32*::*LEU2* This study

JJTY11279 *Mat***a** *his3*Δ*1* *leu2*Δ*0 ura3*Δ*0* *lys2*Δ*0* [pRS316-*ADH1p-SEC7-iRFP*::*URA3*] This study

JJTY11280 *Mat***a** *his3*Δ*1* *leu2*Δ*0 ura3*Δ*0* *lys2*Δ*0* [pRS316::*URA3*] This study

JJTY11580 *Mat***a** *his3*Δ*1 leu2*Δ*0 ura3*Δ*0 met15*Δ*0 gga1*Δ::*KanMX6 GGA2-mCherry*::*URA3* This study
